# Translational Modeling of Psychomotor Function in Normal and AD-Pathological Aging With Special Concerns on the Effects of Social Isolation

**DOI:** 10.3389/fragi.2021.648567

**Published:** 2021-03-19

**Authors:** Lidia Castillo-Mariqueo, Lydia Giménez-Llort

**Affiliations:** ^1^Institut de Neurociències, Universitat Autònoma de Barcelona, Barcelona, Spain; ^2^Department of Psychiatry and Forensic Medicine, School of Medicine, Universitat Autònoma de Barcelona, Barcelona, Spain

**Keywords:** traslacional neuroscience, Alzheimer disease, psychomotor function, motor performance, gait, frailty, isolated, COVID-19

## Abstract

One year after the start of the COVID-19 pandemic, its secondary impacts can be globally observed. Some of them result from physical distancing and severe social contact restrictions by policies still imposed to stop the fast spread of new variants of this infectious disease. People with Alzheimer's disease (AD) and other dementias can also be significantly affected by the reduction of their activity programs, the loss of partners, and social isolation. Searching for the closest translational scenario, the increased mortality rates in male 3xTg-AD mice modeling advanced stages of the disease can provide a scenario of “naturalistic isolation.” Our most recent work has shown its impact worsening AD-cognitive and emotional profiles, AD-brain asymmetry, and eliciting hyperactivity and bizarre behaviors. Here, we further investigated the psychomotor function through six different psychomotor analysis in a set of 13-month-old 3xTg-AD mice and their non-transgenic counterparts with normal aging. The subgroup of male 3xTg-AD mice that lost their partners lived alone for the last 2–3 months after 10 months of social life. AD's functional limitations were shown as increased physical frailty phenotype, poor or deficient psychomotor performance, including bizarre behavior, in variables involving information processing and decision-making (exploratory activity and spontaneous gait), that worsened with isolation. Paradoxical muscular strength and better motor performance (endurance and learning) was shown in variables related to physical work and found enhanced by isolation, in agreement with the hyperactivity and the appearance of bizarre behaviors previously reported. Despite the isolation, a delayed appearance of motor deficits related to physical resistance and tolerance to exercise was found in the 3xTg-AD mice, probably because of the interplay of hyperactivity and mortality/survivor bias. The translation of these results to the clinical setting offers a guide to generate flexible and personalized rehabilitation strategies adaptable to the restrictions of the COVID-19 pandemic.

## Introduction

The COVID-19 pandemic is causing high morbidity and dramatic mortality worldwide. Unfortunately, it has also put pressure on healthcare systems and altered our lifestyles, leading to many worrisome secondary impacts (Brown et al., 2020). Severe measures to curb its spread have been adopted and are still implemented in the new waves, restricting physical and social contact between people, with the elderly population being among the most affected (El Haj et al., 2020). Consequently, preventive strategies and therapeutical interventions for older people, such as promoting social activities, physical and environmental stimulation critical for those with dementia, have been kept to a minimum (Canevelli et al., 2020).

Physical activity is essential to control symptoms and risk factors for many diseases (Warburton and Bredin, 2017). The closures of gyms, swimming pools, and exercise clubs, in addition to laws limiting access to outdoor space and free movement, have inevitably reduced opportunities to exercise or play sports. Decreased physical activity levels in many people may increase other unhealthy lifestyles, but it is also triggering a worsening of the clinical symptoms of diseases, such as Alzheimer's disease (Lautenschlager et al., 2008; Abate et al., 2020; Lara et al., 2020). Exercise is essential to reduce sarcopenia, falls, and fall-related injuries in healthy older adults. Also, the cognitive, cardiorespiratory, and musculoskeletal benefits will be directly affected by the cessation of its performance (Palmer et al., 2020). The closure of day centers has left those whose fragility requires permanent rehabilitation programs at home. Therefore, unprofessional home care may not be enough to meet complex diseases' needs and demands (Wang et al., 2020).

Alzheimer's disease is a complex neurodegenerative disease that leads not only to hallmark cognitive impairment but also to psychomotor dysfunction (O'Leary et al., 2020). Thus, it is one of the leading causes of disability and dependency among older people worldwide. Due to their cognitive and functional deficits, AD patients are vulnerable during crises, especially during the COVID-19 pandemic, and confinement seems to affect neuropsychiatric symptoms in AD patients with low baseline cognitive function (Boutoleau-Bretonnière et al., 2020). It can be overwhelming for those affected and for their caregivers and families, with very high pressure on the direct and indirect healthcare costs (Wang et al., 2020). In the current scenario, people with AD are a particularly vulnerable population due to their complex cognitive and psychomotor dysfunction (Verlinden et al., 2014). Memory problems enhance their difficulties in understanding what is happening (Lara et al., 2020; Wang et al., 2020). This pandemic further exacerbates their vulnerability due to morbidity and mortality from the virus and the pandemic's indirect effects on the health system and support networks on which they depend (Brown et al., 2020). Some studies have reported alterations and exacerbation of cognitive and behavioral symptoms related to confinement and its effects on AD. Worsening of cognitive symptoms, particularly of memory and orientation abilities, the appearance of alterations, such as agitation-aggression, apathy, and depression, the most practical manifestations, have been detected (Boutoleau-Bretonnière et al., 2020; El Haj et al., 2020; Palmer et al., 2020).

Therefore, despite the main clinical characteristic of Alzheimer's disease is cognitive decline and impairment, motor disorders, such as bradykinesia, extrapyramidal stiffness, and gait disturbances are also significant. They will also be affected by the limitations and restrictions dictated to contain and prevent the COVID-19 pandemic (Abate et al., 2020). More excellent knowledge of these psychomotor dysfunctions will contribute to improving the actions to intervene on these deficiencies and impediments that restrict the independence and autonomy of people and their environment.

Like what happens in patients with AD, different mouse models mimic psychomotor deficiencies on a translational level. These deficiencies indicate disease progression when they increase in severity (Buchman and Bennett, 2011; Wagner et al., 2019), making them an essential phenotype for the study of AD progression (O'Leary et al., 2018). The 3xTg-AD model (Oddo et al., 2003) has been widely studied for the impact of Aβ and tau at different study levels, from synaptic plasticity to behavior (España et al., 2010). It mimics various AD symptoms in a temporal and neuroanatomical pattern similar to that observed in humans (Belfiore et al., 2019). After 12 months, a neuropathological profile corresponding to the disease's advanced stages can be observed (Oddo et al., 2003; Belfiore et al., 2019). Thus, this model has made it possible to carry out numerous basic research studies to know the factors related to the progression of the disease as well as preclinical investigations that seek to verify the effect of preventive and therapeutic therapies (Martini et al., 2018).

Therefore, the current study aimed to explore the psychomotor performance of 13-month-old male NTg and 3xTg-AD mice corresponding to normal aging and advanced stages of Alzheimer's disease. We have used a battery to evaluate six different psychomotor functions: spontaneous gait analysis, muscle strength, motor performance, the physical phenotype of frailty. Also, we assessed the impact of isolation in a subgroup of male 3xTg-AD mice that lost their partners and, after 10 months of social life, lived alone for the last 2–3 months.

## Materials and Methods

### Animals

A total of forty-six homozygous 3xTg-AD (*n* = 31) and non-transgenic (NTg, *n* = 15) male mice of 13 months of age in a C57BL/6J background (after embryo transfer and backcrossing of at least 10 generations) established at the Universitat Autònoma de Barcelona (Baeta-Corral and Giménez-Llort, 2014) were used in this study. The 3xTg-AD mice harboring transgenes were genetically modified at the University of California at Irvine, as previously described (Oddo et al., 2003). Animals were kept in groups of 3–4 mice per cage (Macrolon, 35 × 35 × 25 cm) filled with 5 cm of clean wood cuttings (Ecopure, Chips6, Date Sand, UK; uniform cross-sectional wood granules with 2.8–1.0 mm chip size) and nesting materials (Kleenex, Art: 08834060, 21 × 20 cm, White). In the current work, 7 of the 31 3xTg-AD mice had lost their cage mates and lived alone in their cage for 2–3 months. In all cases, standard home cages covered with a metal grid allow the perception of olfactory and auditory stimuli from the rest of the colony. All animals were kept under standard laboratory conditions of food and water ad lib, 20 ± 2°C, 12 h light cycle: dark with lights on at 8:00 a.m. and 50–60% relative humidity.

### Behavioral Assessment

Psychomotor behavior was measured in a behavioral battery consisting of six consecutive steps: (1) Physical Frailty Phenotype, (2) Spontaneous Gait Phenotype: Exploratory activity and (3) Quantitative parameters of gait), (4) Muscular Strength: Forelimb Grip Strength and muscular endurance—Hanger test, (5) Motor performance: Learning, Physical Endurance, and Coordination—Rotarod, and (6) Hindlimb clasping and Geotaxis. Assessments were performed under dim white light (20 lx) during the light cycle of the light cycle: dark (10 a.m. to 1 p.m.). Behavioral evaluations were carried out in 3 days and a counterbalanced manner by observing two independent observers blind to the genotype. The tests were carried out during the morning; 30 min were assigned to habituate the animals in the test room before starting the measurements. We made the following distribution: Day 1—Physical frailty phenotype (body weight, kyphosis, alopecia, etc.) and 2 h later, it was done spontaneous gait analysis; Day 2—Muscle strength and Motor performance; Day 3—Hindlimb clasping and geotaxis. All procedures followed the Spanish legislation on “Protection of animals used for experimental and other scientific purposes” and the EU Directive (2010/63/EU) on this issue. The study complies with the ARRIVE guidelines developed by the NC3Rs and aims to reduce the number of animals used (Kilkenny et al., 2010).

#### Physical Frailty Phenotype

Physical signs of frailty were identified through a physical phenotype that includes the following measurements: body weight, body position, palpebral closure, piloerection, alopecia, tail position, tremor, and kyphosis. These measurements were made before the different tests that are described later. A score of 0 was assigned for normal aspects or 1 for abnormal aspects. Besides, a photographic record was taken of each animal to demonstrate these physical aspects. Also, were measured geotaxis and Hindlimb clasping. Geotaxis was measured using a 10 × 12 cm grid; the time it took for the animal to reach the vertical position from an inverted position at a 90° angle on the grid was recorded in a single trial. Hindlimb clasping closure is a marker of disease progression and severity in several neurodegeneration models in mice. We have included the test described by Chou et al. (2008) and illustrated by Guyenet et al. (2010), which consists of holding the mouse by the tail near its base, observing the hindlegs' position for 10 s in three trials. If the hindlegs are extended continuously outward, away from the abdomen, it is scored with a 0, indicating normality. If one or both hindlegs are retracted toward the abdomen for more than 5 s, a score of 1 and 2 are assigned, respectively. If its hind legs are fully retracted and touching the abdomen for more than half the time, a score of 3 is assigned, indicating greater severity. After each test, the animal is given 30 s of rest.

#### Spontaneous Gait Phenotype

To assess spontaneous gait, the mice were placed in a 27.5 × 9.5 cm transparent test box and observed during a total period of 2 min.

##### Exploratory Activity

In trial 1, the latency to start the movement (taking as reference the movement of the hind legs), the number of explorations (visited corners), the latency and the number of rearing were recorded. Bizarre behaviors were identified during the execution of the walk and classified according to our previous work (Baeta-Corral and Giménez-Llort, 2014). **Figure 2C** shows the path of the circling trajectory of a representative animal. During the tests, defecation and urination were also recorded.

##### Quantitative Parameters of Gait

Two 1-min trials were performed, and gait was recorded by video recording from the undersurface (Cheng et al., 1997). The KINOVEA 8.26 free software was used to identify the metacarpal and metatarsal fore and hind legs and perform the analysis. The quantitative parameters were those described by Wang et al. (2017). A representative animal is illustrated in **Figures 2A,B**.

#### Muscular Strength: Forelimb Grip Strength and Muscular Endurance—Hanger Test

The forelimbs' muscular strength was measured using the hanger test, which is based on a mouse's tendency to grasp a grid or bar instinctively when suspended by the tail. The three trials of the test (1 min ITT) allow discriminating grip strength and muscular endurance, according to the suspension times used (Giménez-Llort et al., 2002). In the first and second trials, grip strength is assessed holding on the animal with its front legs for 5 s at the height of 40 centimeters. In the third trial, the animal is suspended for 60 s in a single attempt to assess muscular endurance. A box with sawdust is placed under the animal to protect it from a possible fall in both cases. The bar used is graduated in 5-cm blocks to obtain the distance covered when the animal moves through the bar; the latency and movement distance are recorded.

#### Motor Performance—Rotarod

To assess motor learning, coordination, and endurance training, mice were evaluated on the constant, accelerated, and rocking Rotarod mode (Ugo basile®, Mouse RotaRod NG). The apparatus consists of five 3 cm diameter cylinders, which are suitably machined to provide grip. Six 25 cm diameter dividers make for five lanes, each 5.7 cm wide, enable five mice to be assessed on the rotor simultaneously. The height to fall is 16 cm. The mice were placed on the rod with their back to the experimenter to measure motor learning, and the rod began to accelerate until it reached 10 rpm. The necessary tests were carried out so that each animal was kept at least 60s on the rod with 1 min of rest between each learning trial. To measure the resistance of the animals, we used the protocol described by Brown and Wong (2007) in which the mice are placed on the rotating rod facing in the opposite direction to the movement of the rod, with an acceleration of 0–48 rpm during a test of 6 min maximum. The test includes six trials with a 1-min rest to start each. A single test measured coordination in the device's rocking mode until reaching 20 rpm with 10 revs, and this mode allows rotations in both directions of the rod. In all tasks, the latency achieved by each animal was recorded.

### Statistics

Statistical analyses were performed using SPSS 23.0 software. Results were expressed as the mean ± standard error of the mean (SEM) for each task and trial. The factors were analyzed with ANOVA, MRA, Student's *t*-test, and Chi-square or Fisher's exact test. The magnitude of the association was measured with Bonferroni. Variables that did not have a normal distribution were transformed using a square root to apply the parametric statistical tests. In all cases, *p* < 0.05 was considered statistically significant.

## Results

**Figure 1:**
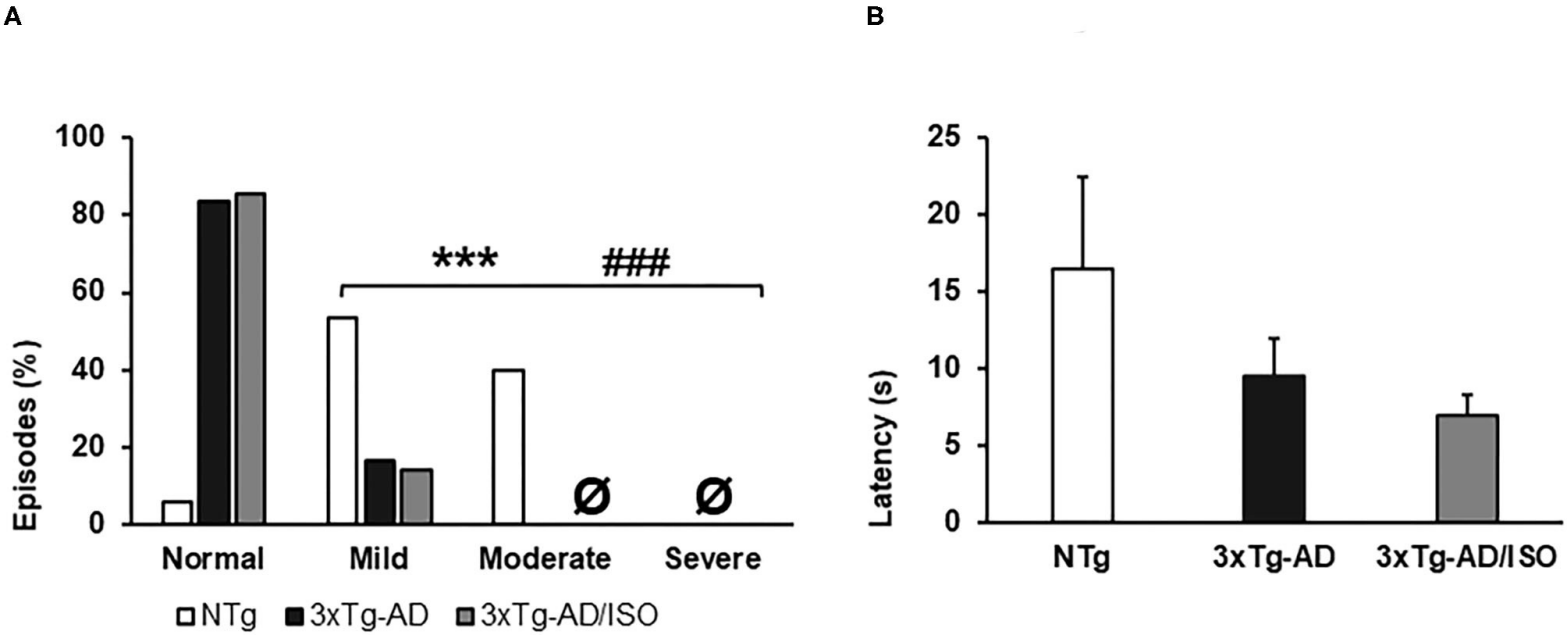
Physical frailty phenotype. **(A)** Hindlimb clasping, the results are expressed as episodes of hindlimb clasping (%). Statistics: X2, *p < 0.05, **p < 0.01, and ***p < 0.001 in 3xTg-AD vs. the NTg group; ^#^*p* < 0.05 and ^##^*p* < 0.01, ^###^*p* < 0.001 in 3xTg-AD/ISO vs. the NTg group. **(B)** Geotaxis, the results are expressed as mean ± SEM. Statistics: One-way ANOVA followed by *post-hoc* Bonferroni test, *p < 0.05, **p < 0.01, and ***p < 0.001 in 3xTg-AD vs. the NTg group; ^#^*p* < 0.05 and ^##^*p* < 0.01, ^###^*p* < 0.001 in 3xTg-AD/ISO vs. the NTg group. **Ø** indicates 0 data in this group.

**Figure 2:**
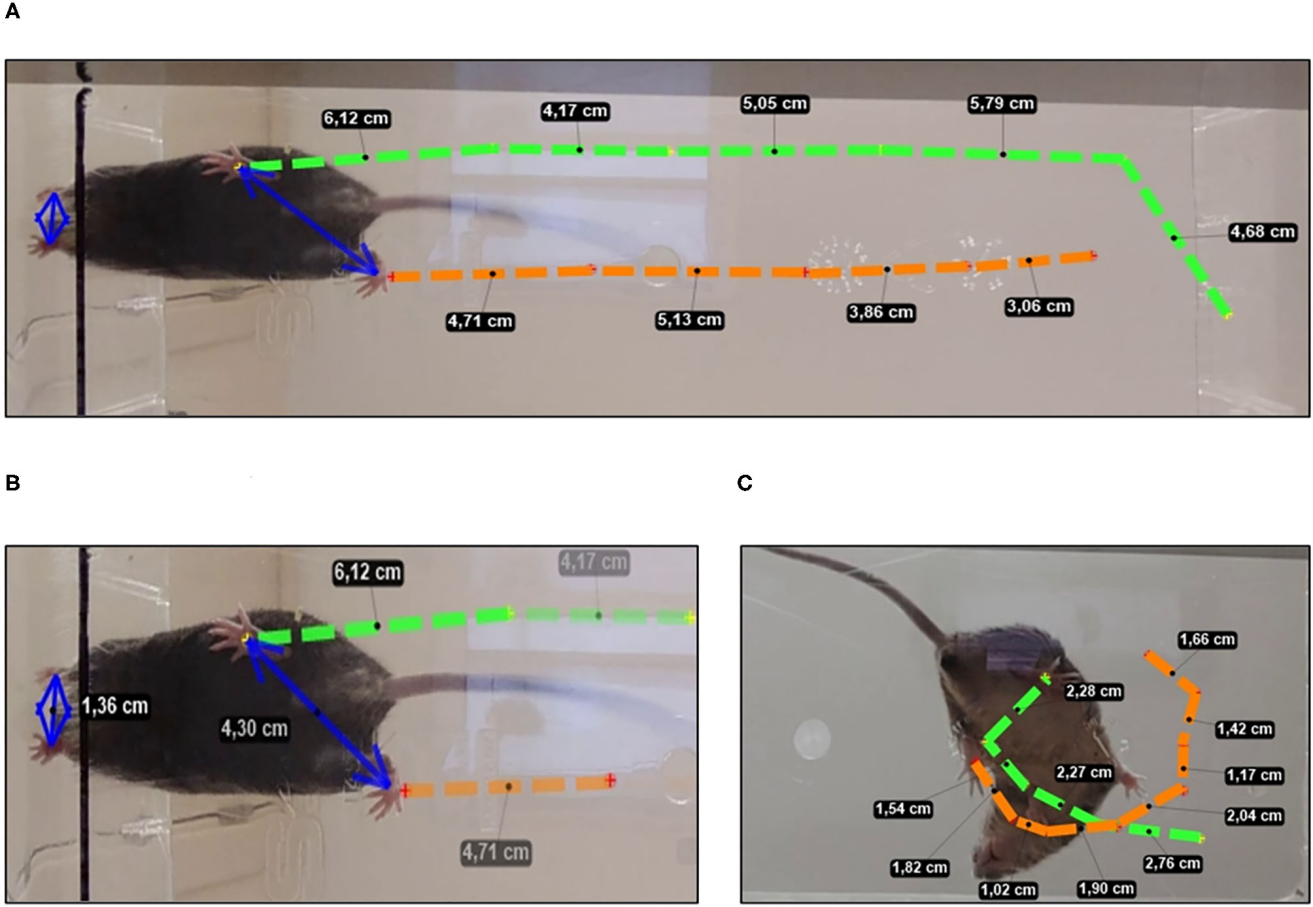
Spontaneous gait analysis. **(A)** Normal gait stride length and **(B)** base of support measurement using hindlimbs and forelimbs paw prints in mice with a normal gait. **(C)** Bizarre circling in a representative 3xTg-AD mice showing a general directional movement, with traced route being short and wide when making narrow circles or having many loops.

**Figure 3:**
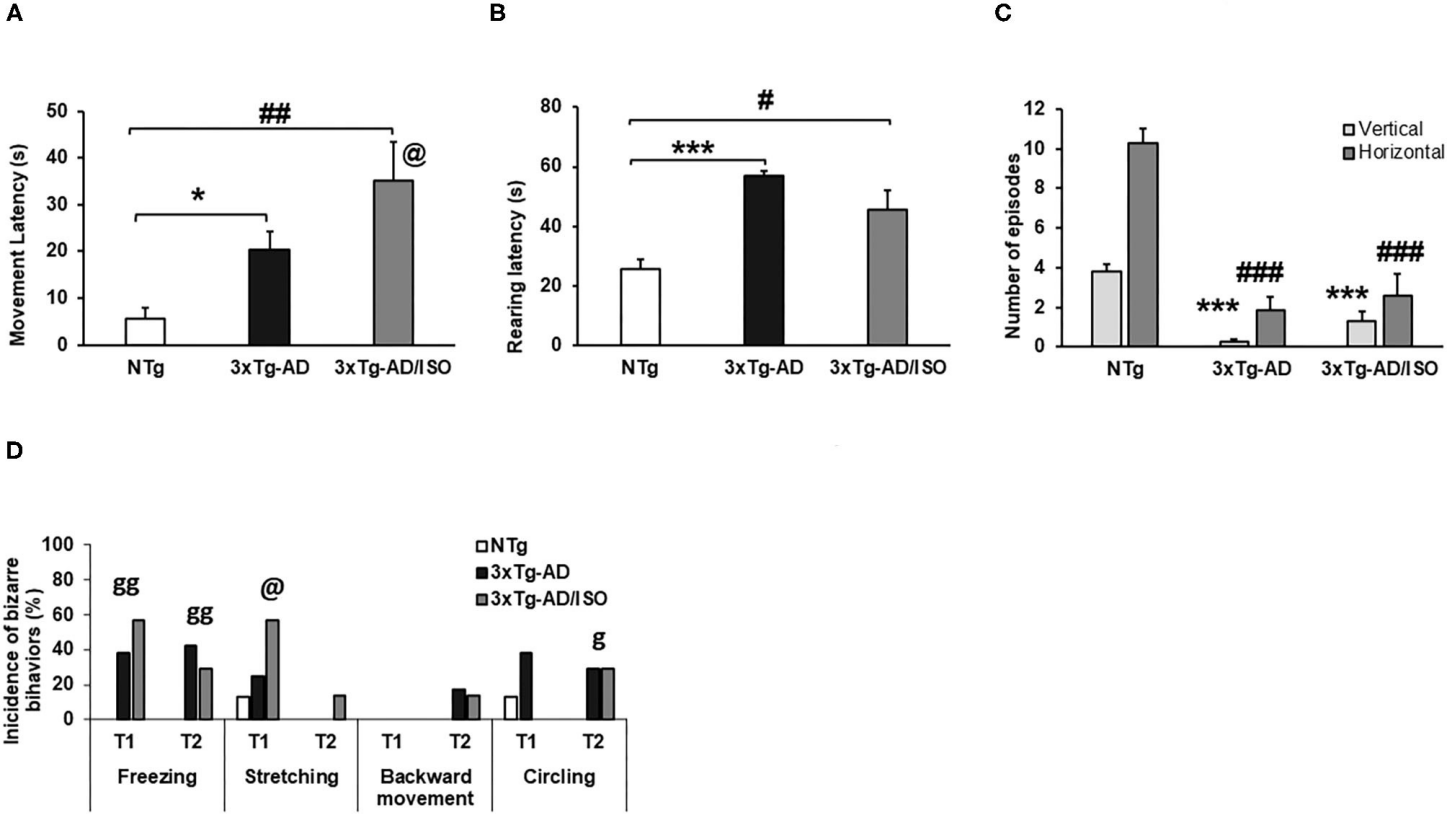
Spontaneous gait phenotype: exploratory activity. Results are expressed as mean ± SEM. **(A)** Freezing (latency of movement); **(B)** Rearing; **(C)** Vertical and horizontal activities. Statistics: One-way ANOVA followed by *post-hoc* Bonferroni test, *p < 0.05, **p < 0.01, and ***p < 0.001 in 3xTg-AD vs. the NTg group; ^#^*p* < 0.05 and ^##^*p* < 0.01, ^###^*p* < 0.001 in 3xTg-AD/ISO vs. the NTg group. Student's *t*-test, 3xTg-AD vs. NTg, in Freezing (Latency of movement), @ indicates isolation. **(D)** Behaviors associated with exploration and circling bizarre behaviors, results are expressed as incidences of bizarre behaviors (%). Statistics: X^2^, ***p < 0.01, **p < 0.05, *p < 0.05, ^n.s.^*p* > 0.05, g indicates genotype, and @ indicates isolation.

**Figure 4:**
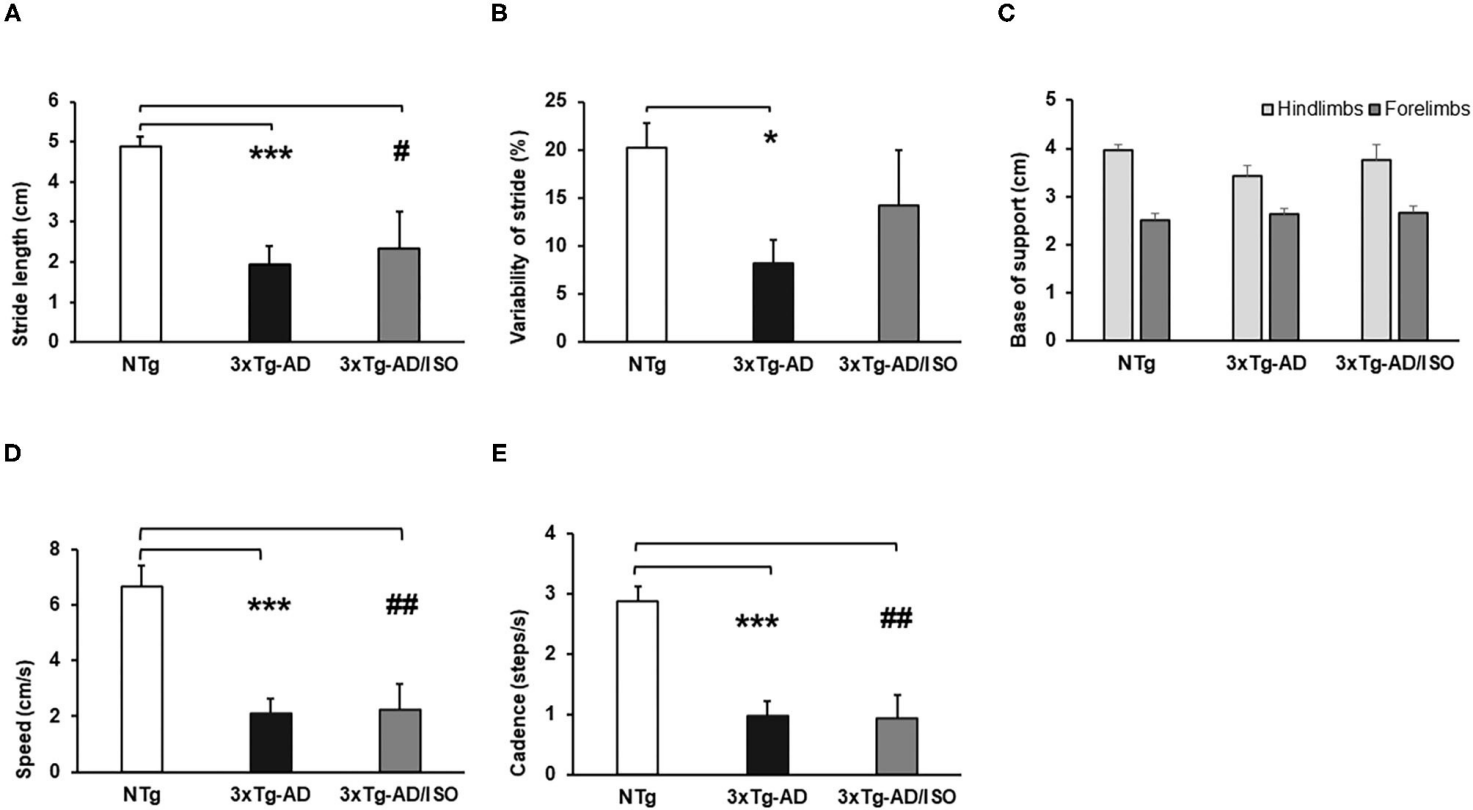
Spontaneous gait phenotype: quantitative parameters of gait. Results are expressed as mean ± SEM. **(A)** Stride Length; **(B)** Variability of Stride Length; **(C)** Base of Support; **(D)** Speed; **(E)** Cadence. Statistics: One-way ANOVA followed by *post-hoc* Bonferroni test, *p < 0.05, **p < 0.01, and ***p < 0.001 in 3xTg-AD vs. the NTg group; ^#^*p* < 0.05 and ^##^*p* < 0.01, ^###^*p* < 0.001 in 3xTg-AD/ISO vs. the NTg group.

**Figure 5:**
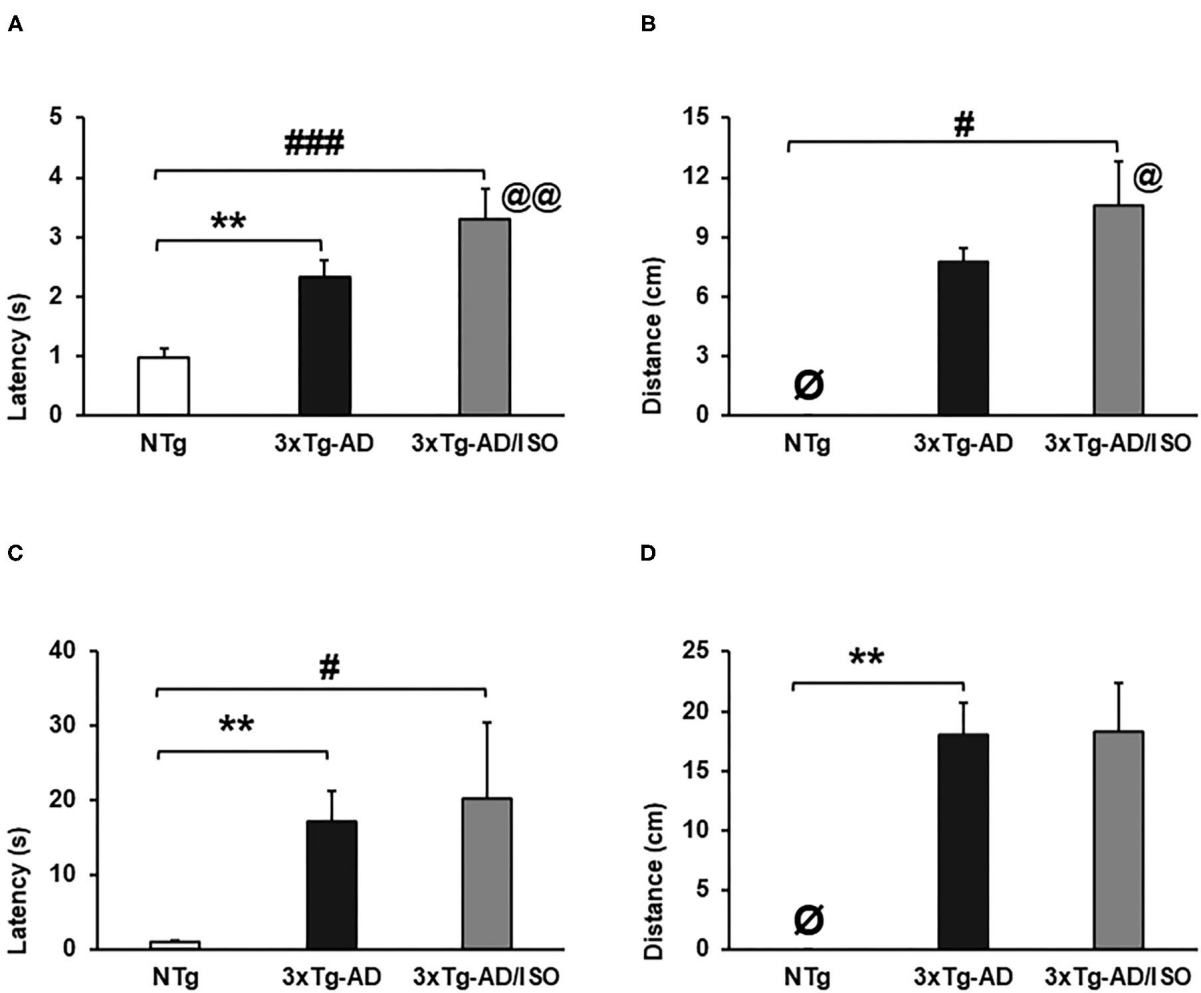
Muscular strength—hanger test. Results are expressed as mean ± SEM. **(A)** Grip Strength-latency; **(B)** Grip-distance; **(C)** Muscular Edurance-latency; **(D)** Endurance-distance. Statistics: One-way ANOVA followed by *post-hoc* Bonferroni test, *p < 0.05, **p < 0.01, and ***p < 0.001 in 3xTg-AD vs. the NTg group; ^#^*p* < 0.05 and ^##^*p* < 0.01, ^###^*p* < 0.001 in 3xTg-AD/ISO vs. the NTg group. Students' *t-*test, 3xTg-AD vs. NTg in Grip Strength-latency and Grip-distance, @ indicates isolation. **Ø** indicates 0 data in this group.

**Figure 6:**
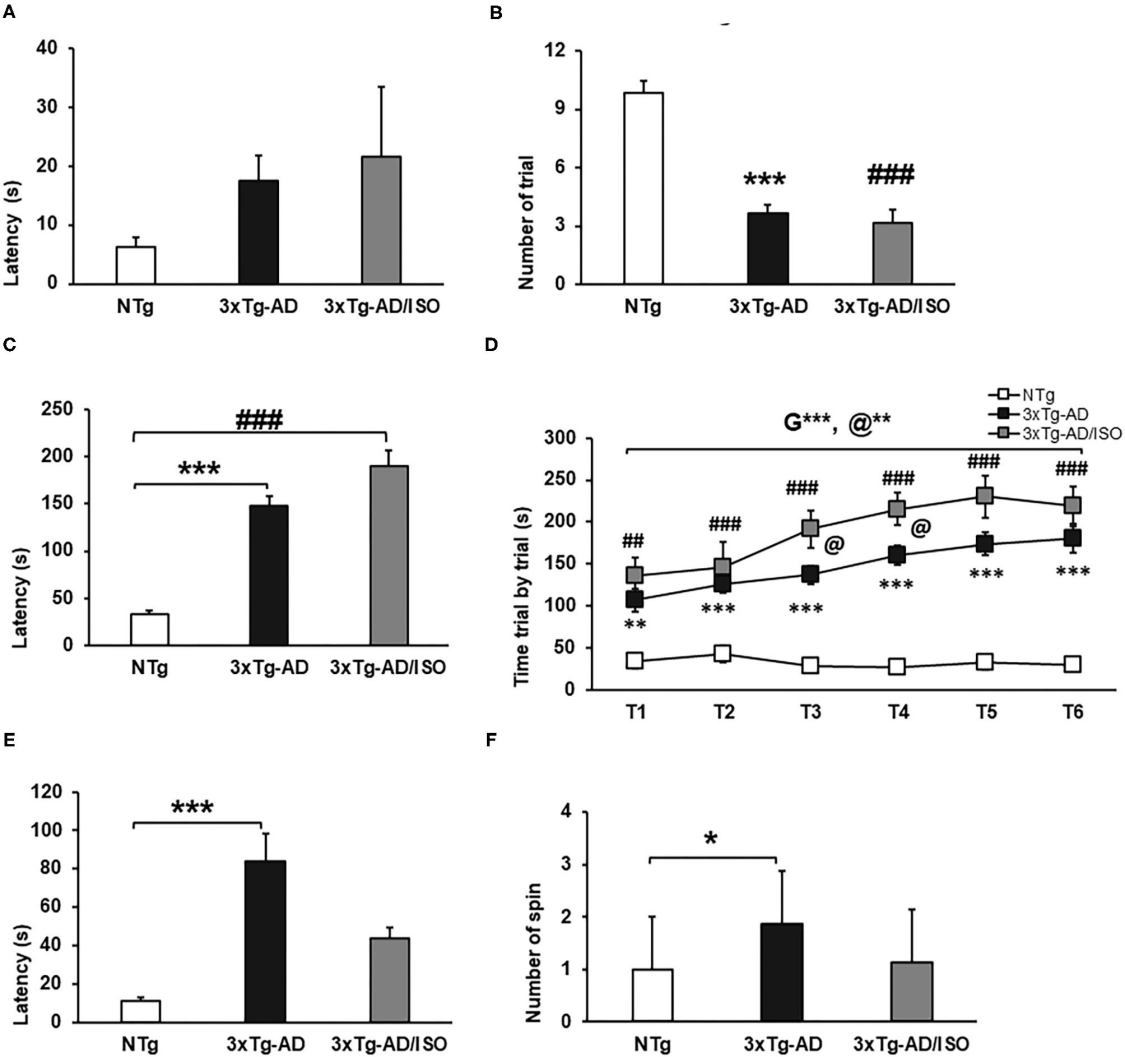
Motor performance—Rotarod. Results are expressed as mean ± SEM. **(A)** Motor learning—Latency; **(B)** Trials learning; **(C)** Physical Endurance-latency; **(E)** Coordination-Latency; **(F)** Spins. Statistics: One-way ANOVA followed by *post-hoc* Bonferroni test, *p < 0.05, **p < 0.01, and ***p < 0.001 in 3xTg-AD vs. the NTg group; ^#^*p* < 0.05 and ^##^*p* < 0.01, ^###^*p* < 0.001 in 3xTg-AD/ISO vs. the NTg group. **(D)** Physical Endurance trial by trial. Statistics: MRA T1-T6, followed by *post-hoc* Bonferroni test, *p < 0.05, **p < 0.01, and ***p < 0.001, G: indicates differences in genotype, @: indicates differences in isolation conditions. One-way ANOVA T1, T2, T3. T4, T5. T6, followed by *post-hoc* Bonferroni test, *p < 0.05, **p < 0.01, and ***p < 0.001 in 3xTg-AD vs. the NTg group; ^#^*p* < 0.05 and ^##^*p* < 0.01, ^###^*p* < 0.001 in 3xTg-AD/ISO vs. the NTg group.

In the first place, we characterized the genotypic differences in psychomotor performance of 13-month-old male 3xTg-AD mice, an age mimicking advanced stages of the disease, compared to age-matched NTg mice with normal aging. Table 1 summarizes the main results obtained, where a clear difference between NTg (*n* = 15) and 3xTg-AD (*n* = 31) mice stands out. To verify our hypothesis that the 3xTg-AD mice that recently lost their home-cage partners exhibited different psychomotor functions, the data of 3xTg-AD mice was depicted in two subgroups, according to their most recent housing conditions. Figures 1–**6** also show the impact of social isolation in the behavior and psychomotor. We analyzed the genotype differences and the effect of isolation according to frailty parameters (see Table 2 and Figure 1). Our results showed that the 3xTg-AD/ISO mice subgroup (*n* = 7) had a high motor performance in physical endurance and muscular strength tests but low performance in exploratory activity and spontaneous gait, considered basic daily life activities.

**Table 1:** Genotype differences between 13-month-old male 3xTg-AD mice and NTg mice in the assessment of psychomotor functions.

**Genotype differences**	**NTg mice *n* = 15 (Mean ± SEM)**	**3xTg-AD mice *n* = 31 (Mean ± SEM)**	**Statistics**
Physical frailty phenotype	(See Table 2 and Figure 1)		
Spontaneous gait **Phenotype: exploratory activity**	(See Figures 3, 4)		
Freezing (latency of movement, s)	5.53 ± 2.38	23.23 ± 2.51	^**^
Rearing (latency, s)	25.61 ± 3.49	52.64 ± 2.69	^***^
Vertical activity (n of counts)	3.80 ± 0.36	0.51 ± 0.16	^***^
Horizontal activity (n of counts)	10.26 ± 0.77	2.03 ± 0.55	^***^
**Quantitative parameters of gait**			
Stride length (cm)	4.88 ± 0.22	2.03 ± 0.39	^***^
Variability of stride length (%)	20.18 ± 2.63	9.60 ± 2.26	^**^
Support base of forelimbs (cm)	2.51 ± 0.12	2.64 ± 0.10	*n.s*.
Support base of hindlimbs (cm)	3.96 ± 0.11	3.48 ± 0.19	*n.s*.
Speed (cm/s)	6.66 ± 0.75	2.13 ± 0.45	^***^
Cadence (steps/s)	2.87 ± 0.24	0.96 ± 0.20	^***^
**Muscular strength: Hanger test**	(See Figure 5)		
Grip strength *(latency, s)*	0.88 ± 0.14	2.55 ± 0.24	^***^
Grip distance *(cm)*	0.0 ± 0.0	2.17 ± 0.73	^*^
Muscular endurance *(latency, s)*	0.92 ± 0.16	17.80 ± 3.87	^**^
Muscular endurance *(distance, cm)*	0 ± 0.0	9.35 ± 2.24	^**^
**Motor performance: Rotarod**	(See Figure 6)		
Motor learning (latency, s)	6.33 ± 1.62	19.51 ± 8.07	^*^
Trials learning (n of trials needed)	9.86 ± 0.61	3.38 ± 0.37	^***^
Physical endurance (latency, s)	32.55 ± 3.83	157.02 ± 9.56	^***^
Coordination (latency, s)	10.93 ± 2.09	74.64 ± 11.80	^**^
Spin (n)	1 ± 0.0	1.70 ± 0.19	^*^
Geotaxis (latency, s)	16.46 ± 5.91	8.89 ± 1.93	*n.s*.

*Student's t-test*,

*** *p < 0.01*,

** *p < 0.05*,

* *p < 0.05*,

*^n.s.^p > 0.05 vs. NTg mice*.

**Table 2:** Physical frailty phenotype.

**Physical frailty phenotype**	**NTg *n* = 15**	**3xTg-AD *n* = 31**	**3xTg-AD *n* =24**	**3xTg-AD/ISO *n* = 7**	**Statistics**
Body weight	43 ± 1.8 g	32 ± 0.1 g	33 ± 0.6 g	32 ± 0.8 g	^***^, ggg
Kyphosis	–	–	–	–	–
Alopecia	3/15 (20%)	9/31 (29%)	5/24 (21%)	4/7 (57%)	@
Body position	–	–	–	–	–
Palpebral closure	–	–	–	–	–
Piloerection	–	–	–	–	–
Tail position	–	4/31 (13%)	4/24 (17%)	–	*n.s*.
Temblor	5/15 (33%)	–	–	–	^***^, gg

*ANOVA; X_2:_*,

*** *p < 0.01*,

** *p < 0.05*,

* *p < 0.05*,

*^n.s.^p > 0.05, g, genotype; @, isolation*.

### Physical Frailty Phenotype

In all groups, the animals had a low prevalence of signs of frailty (Table 2). It must be pointed out that, at this middle age, the NTg group presented overweight [*F*_(2, 45)_ = 25.925, *p* = 0.000 (43 ± 1.8 g)], 33% (5/15) exhibited tremor in the anterior or posterior limbs [X^2^ (df 2), *p* = 0.003] and the sign of hindlimb clasping with a scale of mild and moderate severity [*F*_(2, 45)_ = 31.355, *p* = 0.000; *post-hoc*: 3xTg-AD vs. NTg *p* = 0.000, 3xTg-AD/ISO vs. NTg *p* = 0.000] (Figure 1A). No statistically significant differences were found in the geotaxis, but a trend of transgenic animals performing the test more quickly was noted [NTg = 16.5 ± 5.9; 3xTg-AD = 9.5 ± 2.5; 3xTg-AD/ISO = 7.0 ± 1.3] (Figure 1B). Also, 57% (4/7) of 3xTg-AD/ISO animals presented alopecia in some areas of their body [X^2^ (df 1), *p* = 0.042].

### Spontaneous Gait Phenotype

The sequence of behavioral events developed in the gait test is detailed in Figure 2; the gait analysis of representative animals with normal and bizarre gaits are illustrated in Figure 3, whereas quantitative gait indicators are depicted in Figure 4.

Since the beginning of the test, genotype-dependent differences were found in freezing behavior since it was only present in both groups of transgenic mice [Trial 1, Fisher (df 2), *p* = 0.004; Trial 2, Fisher (df 2), *p* = 0.006]. Backward movement and stretching were also observed. These bizarre behaviors were elicited in greater frequency in transgenic animals, with the most frequent stretching recorded in the 3xTg-AD/ISO group [Trial 1, X^2^ (df 2); *p* = 0.042]. Both types of movements indicate the intention to explore before traveling through a given space (Figure 3D).

On the other hand, the latency to initiate horizontal (freezing latency of movement) in transgenic animals was increased [Freezing (latency of movement), *F*_(2, 45)_ = 7.429, *p* = 0.002; *post-hoc*: 3xTg-AD *vs*. NTg *p* = 0.027, 3xTg-AD/ISO vs. NTg *p* = 0.002]. In this variable, an effect of isolation was shown as a higher motion latency than its group-housed transgenic counterpart [Students' *t*-test*, p* = 0.015] (Figure 3A). In the vertical activity, higher latency was shown in the 3xTg-AD groups compared with other NTg mice [Rearing: *F*_(2, 45)_ = 18.860 *p* = 0.000; *post-hoc*: 3xTg-AD vs. NTg *p* = 0.000, 3xTg-AD/ISO vs. NTg *p* = 0.012] (Figure 3B). Despite this could be due to the presence of freezing, the total vertical (rearings episodes) and horizontal (crossings episodes) exploratory activity was also lower in both groups of transgenic animals compared to the NTg group [N rearing, *F*_(2, 45)_ = 50.400, *p* = 0.000; *post-hoc*: 3xTg-AD vs. NTg *p* = 0.000, 3xTg-AD/ISO vs. NTg *p* = 0,000], [N visited corners, *F*_(2, 45)_ = 36.322, *p* = 0.000; *post-hoc*: 3xTg-AD vs. NTg *p* = 0,000, 3xTg-AD/ISO *vs*. NTg *p* = 0.000] (Figure 3C).

In addition, as illustrated in Figure 4, all the quantitative gait indicators (stride length, variability of stride, speed, and cadence) showed alterations and deficits in displacement and trajectory [Stride length, *F*_(2, 45)_ = 11.552, *p* = 0.000; *post-hoc*: 3xTg-AD vs. NTg *p* = 0.000, 3xTg-AD/ISO vs. NTg *p* = 0.015], [Variability of stride, *F*_(2, 45)_ = 4.714, *p* = 0.014; *post-hoc*: 3xTg-AD vs. NTg *p* = 0.011], [Speed, *F*_(2, 45)_ = 14.393, *p* = 0.000; *post-hoc*: 3xTg-AD vs. NTg *p* = 0.000, 3xTg-AD/ISO vs. NTg *p* = 0.002], [Cadence, *F*_(2, 45)_ = 15,341, *p* = 0.000; *post-hoc*: 3xTg-AD vs. NTg *p* = 0.000, 3xTg-AD/ISO vs. NTg *p* = 0.001] (Figures 4A–E), but not in the anterior or posterior base of support, which remained preserved [Forelimbs: *F*_(2, 45)_ = 0.61, *p* = 0.772; Hindlimbs: *F*_(2, 45)_ = 1.651, *p* = 0.204] (Figure 4C). Increased defecation was recorded in the 3xTg-AD group, reaching 41% of the episodes [X^2^ (df 2); *p* = 0.000].

### Muscular Strength

The assessment of muscle strength of the forelimbs, illustrated in Figure 5, showed a deficit in the NTg control group, while the highest grip strength in the 3xTg-AD/ISO group and the muscular endurance in the 3xTg-AD group. [Grip strength-latency, *F*_(2, 45)_ = 12.958, *p* = 0.000; *post-hoc*: 3xTg-AD vs. NTg *p* = 0.001, 3xTg-AD/ISO vs. NTg *p* = 0.000], [Muscular endurance-latency, *F*_(2, 45)_ = 8.622, *p* = 0.001; *post-hoc*: 3xTg-AD vs. NTg *p* = 0.001, 3xTg-AD/ISO vs. NTg *p* = 0.016] (Figures 5A,C). In the same way, the distance covered by the animal while it was suspended was greater in transgenic animals in the two strength tasks [Grip-distance, *F*_(2, 45)_ = 5.303, *p* = 0.009; *post-hoc*: 3xTg-AD/ISO vs. NTg *p* = 0.008], [Endurance-distance, *F*_(2, 45)_ = 6.113, *p* = 0.005; *post-hoc*: 3xTg-AD vs. NTg *p* = 0.004] (Figures 5B,D).

Additionally, we have detected that the isolated animals have a higher grip and displacement force on this test than their transgenic group-housed counterparts [Grip strength. Students' *t*-test*, p* = 0.007; Grip-distance. Students' *t*-test*, p* = 0.018].

### Motor Performance

In learning and physical endurance tests, transgenic animals' motor performance was higher than that of NTg animals. Transgenic animals needed an average of three trials to learn the test, unlike NTg animals that required an average of nine trials, so the latencies obtained were consequently higher in the transgenic group, albeit they did not reach statistical significance. [Trials learning, *F*_(2, 45)_ = 41.824, *p* = 0.000; *post-hoc*: 3xTg-AD vs. NTg *p* = 0.000, 3xTg-AD/ISO vs. NTg *p* = 0.000] (Figures 6A,B).

Physical endurance, as well as learning, was higher in transgenic animals, with the 3xTg-AD/ISO group being the one who achieved the best physical performance in the six trials. [Physical endurance-latency, *F*_(2, 45)_ = 45.507, *p* = 0.000; *post-hoc*: 3xTg-AD vs. NTg *p* = 0.000, 3xTg-AD/ISO vs. NTg *p* = 0.000] (Figure 6C). [Physical endurance trial by trial, MRA, *F* = 45.515, *p* = 0.000, [ANOVA T1-T6; T1: *F*_(2, 45)_ = 11.304, *p* = 0.001; *post-hoc*: 3xTg-AD vs. NTg *p* = 0.001, 3xTg-AD/ISO *vs*. NTg *p* = 0.000; T2: *F*_(2, 45)_ = 15.791, *p* = 0.000; *post-hoc*: 3xTg-AD *vs*. NTg *p* = 0.000, 3xTg-AD/ISO *vs*. NTg *p* = 0.000; T3: *F*_(2, 45)_ = 35.788, *p* = 0.000 *post hoc*: 3xTg-AD vs. NTg *p* = 0.000, 3xTg-AD/ISO vs. NTg *p* = 0.000; *post-hoc*: 3xTg-AD/ISO *vs*. 3xTg-AD *p* = 0.033; T4: *F*_(2, 45)_ = 54.453, *p* = 0.000; *post-hoc*: 3xTg-AD vs. NTg *p* = 0.000, 3xTg-AD/ISO vs. NTg *p* = 0.000; *post-hoc*: 3xTg-AD/ISO vs. 3xTg-AD *p* = 0.024; T5: *F*_(2, 45)_ = 37.370, *p* = 0.000; *post-hoc*: 3xTg-AD vs. NTg *p* = 0.000, 3xTg-AD/ISO vs. NTg *p* = 0.000; T6: *F*_(2, 45)_ = 30.044, *p* = 0.000; *post-hoc*: 3xTg-AD vs. NTg *p* = 0.000, 3xTg-AD/ISO vs. NTg *p* = 0.000] (Figure 6D).

Regarding the coordination measured in the rotarod, the 3xTg-AD animals exhibited higher latency than the NTg animals, exceeding 60 s in this test. In addition, there was a greater use of spin or postural strategies to stay on the bar while turning in both directions, [Coordination-latency: *F*_(2, 44)_ = 8.786, *p* = 0.001; *post-hoc*: 3xTg-AD vs. NTg *p* = 0,000], [Spin: *F*_(2, 45)_ = 5.461, *p* = 0.008; *post-hoc*: 3xTg-AD vs. NTg *p* = 0.010] (Figures 6E,F).

## Discussion

The present work assessed the psychomotor functions in male mice with normal and AD-pathological aging and the impact of “naturalistic isolation” in a subgroup of 3xTg-AD mice. Male sex was chosen to explore further the stronger sex-dependent motor effects of aging reported in male C57BL/6 mice than in females (Baeta-Corral and Giménez-Llort, 2015) and because at this age, the singularity of the natural isolation scenario only occurs in male 3xTg-AD mice as a result of their neuroimmunoendocrine derangement and increased mortality rates (Giménez-Llort et al., 2008). The results indicated genotype differences with paradox better performance in motor variables involving more significant physical work in 3xTg-AD animals independently of social isolation, and a delayed appearance of motor deficits related to physical resistance and tolerance to exercise in 3xTg-AD mice survivors that remained isolated during 2–3 months. However, in the variables that involve information processing and decision-making to perform a task (exploration and gait), these animals exhibited poor or deficient performance that includes circling as bizarre behavior.

Regarding the physical frailty phenotype, middle-aged NTg animals were obese and presented tremors in the extremities accompanied by partial alopecia in some animals. In the 3xTg-AD mice, the frequency of alopecia was similar to that of NTg animals, accompanied by a high, stiff tail that is associated with alert and arousal in animals. Most 3xTg-AD/ISO animals presented some degree of alopecia in their body, but normal parameters were found in the rest of the variables. Unlike the results obtained by Kane et al. (2018), 3xTg-AD mice were more fragile than NTg males, accompanied by higher mortality. This was in agreement with our recent work in end-of-life scenarios (Muntsant et al., 2021).

On the other hand, geotaxis and buckling may indicate NTg animals' alterations due to body weight, like that observed in rotarod, and lighter mice perform better than heavier mice (Stover et al., 2015). In particular, the grip has been described as an alteration of the extremities' reflexes due to motor coordination deficits, neurological signs that resemble myoclonic movements, epileptic seizures, or pathological reflexes that alter gait (Lalonde et al., 2012). However, we have already shown in male C57BL/6 mice that grip strength and prehensility are also sensitive to body weight and fat composition associated with the aging process (Baeta-Corral et al., 2018).

Frailty and dementia are closely related and share similar common risk factors, such as sociodemographic factors, comorbidities, and lifestyle factors (Buchman et al., 2008; Wallace et al., 2018; Petermann-Rocha et al., 2020). According to the results obtained in human beings in several studies, the decrease in grip strength and a slow gait speed or the deterioration of balance have been attributed to a worse cognitive condition among people with frailty, which contributes to the incidence of dementia, including AD (Li, 2002; Hanlon et al., 2018; Lim et al., 2018; Petermann-Rocha et al., 2020).

In this respect, it is noteworthy that the gait of the 3xTg-AD mice showed deficits similar to gait with an aging pattern, accompanied by a series of bizarre behaviors that can interfere with the trajectory and movement similar to that reported by Muntsant and Giménez-Llort (2020a) in long-term isolation in male mice. At the beginning of the task, a long freezing period is accompanied by a high latency in the exploratory activity that interferes with the horizontal and vertical activity counts compared to the NTg group. This neophobic response corresponds to one of the most sensitive ethological behaviors of the 3xTg-AD phenotype detected in previous studies (Giménez-Llort et al., 2007; Giménez-Llort, 2010; Muntsant and Giménez-Llort, 2020a). Likewise, we can distinguish that the 3xTg-AD group-housed animals presented several episodes in the horizontal and vertical components slightly lower than the 3xTg-AD isolated animals, contrary to what occurs in the movement latency at the beginning of the test, where the isolated animals 3xTg-AD took longer to perform movements, thus influencing the results of all quantitative gait parameters.

Bizarre behaviors are mainly related to psychiatric and neurological disorders (Giménez-Llort et al., 2002; Baeta-Corral and Giménez-Llort, 2014; Cordón-Barris et al., 2016). In previous studies, it has been reported that these behaviors can also be provoked when animals are subjected to unfamiliar environments, mainly those used to evaluate anxiety behavior as a manifestation or response of stress (Willner, 1991; Giménez-Llort et al., 2002, 2007). We described that is at the age of 6 months when the initial freezing response observed in 3xTg-AD and NTg animals of both sexes when assessed under anxiogenic conditions, such as the open field test or the corner test is more likely to be followed by the elicitation of bizarre behaviors (Baeta-Corral and Giménez-Llort, 2014). During the tests, the animals exhibited behaviors considered bizarre that were classified as stereotyped stretching, stereotyped rearing, backward movement, and jumping, apparently without a purpose but considered coping-with-stress strategies. In the Morris water maze, a stressful scenario for mice, we have already reported circling swimming behavior in 6-month-old 3xTg-AD mice (Castillo-Mariqueo and Giménez-Llort, 2019). The presence of this bizarre behavior worse with the progress of the disease as it is a distinctive swimming pattern in male 3xTg-AD mice at 13 months of age, modeling advanced disease stages (Baeta-Corral and Giménez-Llort, 2015). In the present work, the results corroborate that when faced with novelty and recognition of places, 3xTg-AD mice exhibit these behaviors, which delays the appearance of horizontal and vertical exploration. Stretching and circling were behaviors exhibited by isolated animals, suggesting that stretching behavior or risk assessment was sensitive to social conditions.

In the quantitative gait parameters, 3xTg-AD mice showed deficits like gait with an aging pattern, accompanied by a series of bizarre behaviors that can interfere with trajectory and movement, as mentioned above. The gait analysis reported by Brown's laboratory in 16-month-old animals did not show significant differences in the length or width of the stride between genotypes or sexes (Garvock-de Montbrun et al., 2019), whereas at 6 months of age, the animals exhibited a longer stride than NTg mice (Stover et al., 2015). In the present work, we have detected a decrease in all variables related to stride length in the transgenic group (cadence, speed, and stride variability) without altering the base of support of the front and rear extremities are similar to those detected in the control group.

Gait disorders in patients with AD have been described within the group of disorders known as “frontal gait,” and in particular, gait in AD has been defined as “cautious gait” (Pirker and Katzenschlager, 2017; Baker, 2018). At the same time, cautious gait occurs more frequently in patients with mild dementia (Clinical Dementia Rating Scale: Hughes CDR, stage 1). This gait pattern is like the one observed in aging, and it may present a decrease in speed, stride length, and gait postural stability, which is manifested more specifically in static and dynamic balance, with a widened support base (Scherder et al., 2007). Dynamic instability has also been observed in mild and moderate AD (Mesbah et al., 2017). In advanced AD stages, the disorder becomes more prominent, and the gait has been described as “frontal gait.” At this stage, the person has difficulties standing up and postural maladjustments that prevent the change to different positions in coordination with the segments, such as arms and legs, causing difficulties to achieve a stable position (Munoz et al., 2010; Pirker and Katzenschlager, 2017). It is complex to mimic human motor disorders in mice, but we have detected some similarities in gait execution. The onset of gait in humans is affected as occurs in Parkinson's disease, with bradykinesia, some patients try to start gait by swinging the trunk laterally or by exaggerated movements of the arms, there is dragging of the feet, but it disappears after walking a few steps with what the gait usually improves (Beauchet et al., 2016; Montero-Odasso and Perry, 2019). Also, freezing or freezing episodes can occur, especially when turning and when facing obstacles (Muir et al., 2012). Although AD's clinical feature is declining cognition, the motor signs that frequently accompany AD often precede and predict AD's clinical diagnosis (Munoz et al., 2010). In 3xTg-AD animals, the bizarre behaviors described above appear to be a translational approach to detecting the severity of the psychomotor disorders presented in Alzheimer's disease, and they differentiated the effects of social isolation.

We have found significant differences in muscular strength that indicate that 3xTg-AD animals have a conserved strength in isolation, and this is the first time this finding has been reported. Already at 6 months of age, Stover et al. (2015) reported that the 3xTg-AD mice had a lower strength than the NTg mice at 16 months, Garvock-de Montbrun et al. (2019) found no significant differences, although the 16-month-old mice were heavier than those of 6 months, his grip strength did not decrease.

It has been previously reported that in humans, there is an association between the pathology of AD in the cognitive regions and the grip strength or grip strength (Buchman et al., 2008; Boyle et al., 2009). Also, the loss of strength and muscle mass is frequent in the aging; even BMI and frailty are associated with AD's risk (Boyle et al., 2009; Moon et al., 2019). Thus, muscle mass and strength are not related to each other in the male and female groups. In AD patients, large muscle mass does not mean more significant power. A simple assessment of lower extremity muscle strength is effectively predicted cognition than mass muscle measurement in male patients (Moon et al., 2018, 2019). Stover et al. (2015) reported that 6-month-old 3xTg-AD mice had a higher motor performance in both strength and rotarod tests than their NTg counterparts. In the current 13-month-old NTg animals studied, we cannot rule out that body weight may be the variable that modifies muscle strength as this group is made up of obese animals, so it is necessary to contrast these results with normal-weight animals.

The motor performance performed by the isolated 3xTg-AD animals is even higher than the 3xTg-AD grouped animals, which has allowed us to discriminate its effect in this group. At the clinical level, exercise is a widely used therapeutic resource to contribute to the treatment of the disease's symptoms and improve patients' quality of life with AD (Dao et al., 2013; Meng et al., 2020). Physical exercise is related to maintaining an optimal cognitive state and adequate maintenance of the mainly musculoskeletal and cardiovascular systems, making it a protective factor of health (Fielding, 1995; Taylor, 2014; Langhammer et al., 2018). In these animals, the practice of exercise during the six trials studied shows an increase in physical resistance as the test develops. This variable may indicate that the basal physical state of these animals is optimal and that the effect of physical exercise enhances their performance. We can highlight that although the rotarod results indicate that mice improved motor performance, they performed worse in other tasks that relate to cognitive and affective variables, in agreement with the hallmarks of the disease.

On the other hand, the 3xTg-AD animals in the coordination test carried out in the rotarod reached a higher performance without isolation effects. This group managed to stay on the rotating bar for more than 1 min, performing an average of two turns, reflecting the postural adjustment necessary to avoid falling from the bar. In contrast, in humans, it has been shown that alterations in balance and coordination are clinically demonstrable in people with mild cognitive impairment and AD (Franssen et al., 1999). These findings indicate that balance control aspects deteriorate with increasing severity of cognitive impairment and that executive function plays an essential role in controlling balance and coordination (Franssen et al., 1999; Eggermont et al., 2010; Tangen et al., 2014).

One of the main differences concerning the genotype NTg group may be due to changes typical of aging. We know that there is a functional decline as age increases. Also, there is a slow and gradual sensory deterioration (Cavazzana et al., 2018). Studies of auditory, visual, and vestibular sensory deficits and alterations in the C57BL/6 strain suggest a deterioration in these systems, leading to functional and cognitive deterioration in this group of animals. As a result, the impaired sensory system could induce poor performance in some of the animals' tests (Shiga et al., 2005; Vijayakumar et al., 2015). Besides, the obesity present in animals can be interference to achieve optimal performance in some tests, for example, those related to more excellent work of physical resistance and muscular strength and deficits and alterations, such as tremor together with hindlimb clasping. Some studies indicate that this strain tends to develop severe obesity if put on a high-fat diet (Brownlow et al., 1996; Williams et al., 2003). Other studies point to obesity in C57BL/6J animals as one of the changes associated with aging, in which the increase in adipose tissue alters energy metabolism and cardiovascular function (Krishna et al., 2016; Chu et al., 2017). Here, it is interesting to note that in previous work, we have shown that 6-month-old C57BL/6 with the same bodyweight but higher fat composition due to d-galactose-induced accelerated-aging exhibited reduced equilibrium, muscular strength, coordination, and prehensility, and these effects were only found in male sex (Baeta-Corral et al., 2018). In that work, the effects of accelerated aging on balance, motor coordination, and learning were also tested on an accelerating rotarod showing differences in the number of training trials needed to learn to walk on the lane, and the distance traveled once the task was learned.

Social isolation, from this perspective of social deprivation, would increase the vulnerability to stress episodes (Bartolomucci et al., 2003). Studies carried out in different mouse models (Swiss CD-1, Tg2576, 3xTg-AD) point out that individually housed mice show a reduced neophobic reaction and decreased anxiety compared to group-housed mice (Dong et al., 2004, 2008; Rothman et al., 2012). Furthermore, an anxious animal shows a more significant latency to explore the novel environment (Palanza et al., 2001). Bartolomucci et al. (2003) pointed out that when Swiss CD-1 mice are challenged with a new stimulus, individually housed mice respond with less fear and more extraordinary exploration and locomotion than group-housed mice. Similarly, we have recently shown that naturalistic isolation in 3xTg-AD elicited hyperactive patterns, as measured in both gross and fine-motor functions (Muntsant and Giménez-Llort, 2020a). Pathophysiology is critical to differentiate the underlying mechanisms that trigger these responses. In the present study, animals were left alive for monitoring until the more advanced ages of life, and therefore, the impact on the HPA axis and neuropathology could not be determined. However, in our precedent work using the same “naturalistic isolation” approach, isolated 3xTg-AD mice showed increased AD-brain asymmetry in the hippocampus and cortical areas, and the above mentioned behavioral alterations were correlated to increased hippocampal tau pathology (Muntsant and Giménez-Llort, 2020a,b). Previous work in these findings in the literature suggests that 3xTgAD mice are more vulnerable than control mice to chronic psychosocial stress, resulting in an exacerbation of Aβ accumulation and impairs neurotrophic signaling (Rothman et al., 2012). On the other hand, Tg2576 mice exhibit increases in plasma corticosterone and increases in the expression of GR and CRFR1 in the cortex and hippocampus, in association with increases in the level of Aβ in brain tissue, plaque deposition of Aβ, and atrophy of the hippocampus (Dong et al., 2008).

In summary, we found standard and distinctive psychomotor features between the normal and pathological aging AD samples and the impact of the social isolation scenario. We can highlight the genotype factor and physical activity level as a protective mechanism, although physical frailty phenotype indicators are present. While the 3xTg-AD mice showed more significant deterioration in the physical aspects, their motor learning capacity remained preserved. Additionally, these animals exhibited higher performance in exercise tolerance and muscle strength tests, where the genotype seems to be a determining factor in general performance. On the other hand, the “naturalistic isolation” studied here seemed to interfere with motor performance. The presence of freezing at the beginning of the exploratory activity and spontaneous gait test was associated with increased functional limitation in this group. On the contrary, the physical parameters: strength, and physical performance in rotarod, apparently are not altered, showed a coincidence with hyperactivity or anxiety, one of the manifestations of the advanced stages of AD.

These findings generate new hypotheses to study the underlying biological mechanisms and have been useful to be applied in translational scenarios of geriatric rehabilitation (Castillo-Mariqueo et al., 2020), where timely geriatric interventions (Giménez-Llort, 2010) should be one of the priorities to counteract the second impact of the current pandemic in the older adults with dementia.

## Data Availability Statement

The raw data supporting the conclusions of this article will be made available by the authors, without undue reservation.

## Ethics Statement

All procedures are approved by CEEAH and are in accordance with Spanish legislation on Protection of Animals Used for Experimental and Other Scientific Purposes and the EU Council directive (2010/63/UE) on this subject. The study complies with the ARRIVE guidelines developed by the NC3Rs and aims to reduce the number of animals used.

## Author Contributions

LG-L: conceptualization. LC-M: performance, analysis of behavior, and illustrations. Both authors equally contributed to the scientific discussions, the writing, and approval of the manuscript.

## Conflict of Interest

The authors declare that the research was conducted in the absence of any commercial or financial relationships that could be construed as a potential conflict of interest.
